# The effect of exercise as an intervention for women with polycystic ovary syndrome: A systematic review and meta-analysis

**DOI:** 10.1097/MD.0000000000019644

**Published:** 2020-04-17

**Authors:** Isis Kelly dos Santos, Maureen C. Ashe, Ricardo Ney Cobucci, Gustavo Mafaldo Soares, Tecia Maria de Oliveira Maranhão, Paulo Moreira Silva Dantas

**Affiliations:** a Health Sciences Postgraduate Program, Federal University of Rio Grande do Norte, Natal, Brazil; b Department of Family Practice, The University of British Columbia; c Centre for Hip Health and Mobility, Vancouver, British Columbia, Canada; d Biotechnology Postgraduate Program and Medicine School, Potiguar University of Rio Grande do Norte; e Department of Gynecology and Obstetrics; f Department of Physical Activity, Federal University of Rio Grande do Norte, Natal, Brazil.

**Keywords:** body composition, exercise therapy, hormones, menstrual cycle, polycystic ovary syndrome

## Abstract

**Background:**

Polycystic ovary syndrome (PCOS) affects reproductive-aged women and is associated with increased prevalence of serious clinical problems including: reproductive implications, metabolic dysfunction, and cardiovascular risk. Physical activity offers several health benefits for women with PCOS. The aim of this systematic review was to synthesize evidence on the effect of different types of exercise on reproductive function and body composition for women with PCOS.

**Methods:**

This was a systematic review and meta-analysis of randomized controlled trials (RCTs) following recommended review methods. We searched 6 databases: Cumulative Index of Nursing and Allied Health Literature; Embase; MEDLINE (*via* Ovid); PubMed; Sport Discus; and Web of Science; and we developed search strategies using a combination of Medical Subject Headings terms and text words related to exercise interventions for women with PCOS. There was no restriction on language or publication year. The search was conducted on April 16, 2019 and updated on November 15, 2019. Two authors independently screened citations, determined risk of bias and quality of evidence with Grading of Recommendations Assessment, Development and Evaluation. We conducted meta-analyses following recommended guidelines, and report results using standardized mean difference (SMD).

**Results:**

Ten RCTs (n = 533) were included in this review. Studies tested the following interventions: aerobic, resistance, and combined (aerobic/resistance) training programs. Most studies were small (average 32, range 15–124 participants), and of relatively short duration (8–32 weeks). There was high heterogeneity for outcomes of reproductive function (menstrual cycle, ovulation, and fertility). We noted low certainty evidence for little to no effect of exercise on reproductive hormones and moderate certainty evidence that aerobic exercise reduced body mass index (BMI) in women with PCOS: BMI SMD −0.35, 95% confidence interval −0.56 to −0.14, *P* = .001.

**Conclusion:**

For women with PCOS, evidence is limited to discern the effect of exercise on major health outcomes (e.g., reproductive function). There is moderate certainty evidence that aerobic exercise alone is beneficial for reducing BMI in women with PCOS. Future studies should be conducted with longer duration, larger sample sizes, and should provide detailed information on menstrual cycle and fertility outcomes.

PROSPERO Systematic review registration: 2017 CRD42017058869.

## 1 Introduction

Polycystic ovary syndrome (PCOS) is an endocrine disorder characterized by changes in hormonal levels. It is associated with increased prevalence of serious clinical problems including: insulin resistance, hypertension, obesity, diabetes mellitus, dyslipidemia, depression, anxiety, cardiovascular risk, and reproductive implications that affect many women of reproductive age.^[1,2]^ The most common reproductive symptoms of PCOS are high production of male hormones, menstrual irregularity, anovulatory infertility, and pregnancy complications.^[3,4]^

High levels of insulin stimulate ovaries to increase androgenic secretion, and have inhibitory effects on the hepatic production of sex-hormone-binding globulin (SHBG), thus insulin resistance can also affect ovulation and consequently increase the risk of infertility.^[5,6]^ Above all, infertility is a major concern for women with PCOS which can result in the inability to conceive.^[7]^ However, lifestyle modification (including physical activity and diet) is recommended as a 1st-line therapy for women with PCOS since lifestyle factors can reduce insulin resistance, and thus improve metabolism and reproductive function.^[8,9]^ Numerous studies demonstrate that weight loss can restore the menstrual cycle and ovulation in women with PCOS, making it an important element to consider in the management of reproductive function.^[8–10]^

Multiple systematic reviews and meta-analyses^[11–14]^ highlight the beneficial effect of exercise for PCOS symptom management. However, discordant and limited findings on exercise characteristics lead to challenges for its prescription for women with PCOS. Although there are systematic reviews of lifestyle interventions in the management of PCOS outcomes, few studies separate exercise from diet.^[15–18]^ Therefore, despite exercise as an important component of nonpharmacologic management of PCOS symptoms, no previous review synthesized evidence for different types of exercise.

Therefore, the objective of this systematic review was to synthesize available evidence on the effects of different types of exercise on reproductive function (major outcomes) and body composition (minor outcomes), in women with PCOS. This practical knowledge can support clinical practice for exercise prescription (e.g., type), and guide the conduct of future studies.

## 2 Methods

Protocol registration: This was a systematic review with meta-analysis of randomized clinical trials on the effect of exercise (by type) on reproductive function of women with PCOS. We followed guidelines for conducting and reporting systematic reviews using Preferred Reporting Items for Systematic Reviews and Meta-analysis (PRISMA).^[19]^ We registered the review on PROSPERO (2017 CRD42017058869), and provided updates to the protocol, when appropriate. Our review question was: For women with PCOS, what is the effect of exercise and type of exercise on: major outcomes: menstrual cycle, hormonal levels, ovulation rate (reproductive function); and minor outcomes: metabolic parameters (HOMA-IR), and body composition (weight (kg), body mass index (BMI), waist circumference (cm), and waist hip ratio.

Systematic review study team members: The team was composed of 6 members including experts in women's health, exercise physiology, physical activity, and methods related to conducting systematic reviews.

*Eligibility criteria (concepts):* We identified peer-reviewed publications that included the following criteria: population: women (18–40 years) diagnosed with PCOS based on the Rotterdam criteria, National Institutes of Health criteria or who present menstrual dysfunction and infertility; intervention: We only included randomized controlled trials (RCTs) that tested exercise, a *“*subset of physical activity that is planned, structured and repetitive and has as a final or an intermediate objective the improvement or maintenance of physical fitness” (p. 128),^[20]^ which was at least 8 weeks in duration; comparator: We did not restrict inclusion by type of comparator; however, the trial needed to include an exercise only arm; outcome: reproductive function (hormones, menstrual cycle, ovulation rate [major], metabolic parameters, and body composition [minor]). We excluded trials with adolescents (mean age <18 years of age) and animal studies. The search was conducted on April 16, 2019 and updated on November 15, 2019.

*Information sources and searches:* We included all peer-reviewed publications of RCTs, regardless of language or year of publication. We searched the following databases: Cumulative Index of Nursing and Allied Health Literature; Embase; MEDLINE (*via* Ovid); PubMed; Sport Discus; Web of Science; and Google Scholar (advanced feature). We developed search strategies using a combination of Medical Subject Headings terms and text words related to exercise interventions for women with PCOS. We provide an example of our search strategy in Table 1.

**Table 1:**
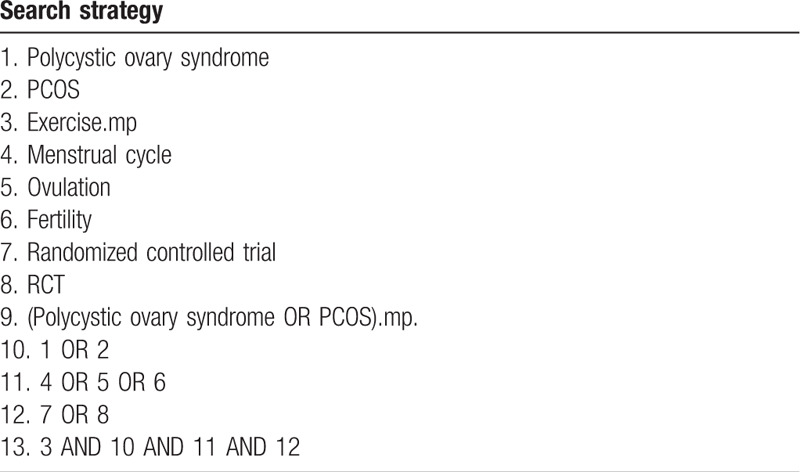
MEDLINE (Ovid) search strategy.

**Figure 1:**
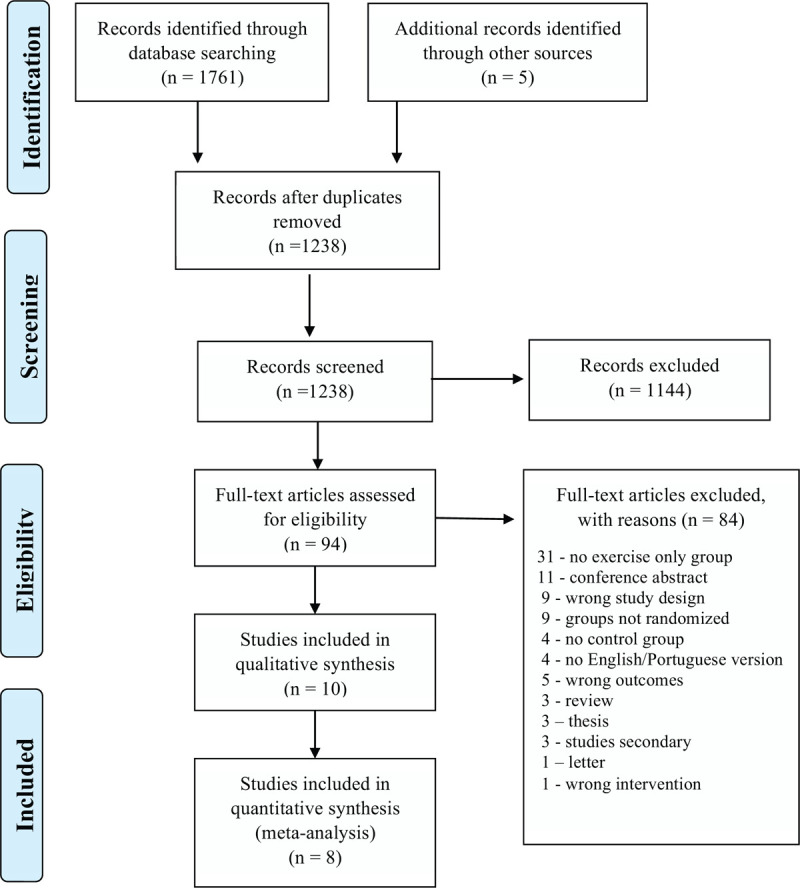
PRISMA flow diagram for the systematic review.

*Study selection (screening, level 1, level 2):* We used Covidence (Covidence Systematic Review Software; Veritas Health Innovation, Melbourne, Australia) for screening citations at level 1 (title and abstract), level 2 (full text), extracting data, and adjudicating risk of bias. Two authors (IKS, MCA) initially independently reviewed each article based on title and abstract (level 1). After this step, the same authors independently evaluated the full text of the selected articles following the inclusion criteria (level 2). The final decision on the inclusion of studies was decided through consensus, or by a 3rd author (TMOM). We documented reasons for exclusion at level 2 only, selection process followed PRISMA flow diagram (Fig. 1).

*Data extraction process:* One author (IKS) extracted study characteristics, and a 2nd author (PMSD) confirmed data. When related studies had several publications with the same participants (but different outcomes), we included the main study, and extracted additional details from related publications.

*Data synthesis and analysis:* We used Review Manager (RevMan 5.3; Cochrane Collaboration), for data analyses and to generate figures, following standard guidelines.^[21]^ We evaluated heterogeneity between studies through discussion and the *I*^2^ statistic (<25%, low heterogeneity, 25–50%, moderate heterogeneity, and >50%, high heterogeneity).^[22]^ For continuous outcomes, we used standardized mean difference (SMD) with 95% confidence interval (CI) and random-effects models.^[23,24]^ For studies with 2 or more arms of the same exercise type (aerobic exercise), we combined the interventions in the meta-analysis using standard procedures based in Cochrane Handbook.^[21]^ For major and minor outcomes, we assessed the certainty of evidence according to Grading of Recommendations Assessment, Development and Evaluation (GRADE),^[25,26]^ using GRADE PRO software (https://gdt.gradepro.org). Two reviewers (IKS, RNC) evaluated the quality of evidence using GRADE, and resolved discrepancies by consensus.

*Summary measurements:* Major outcomes were reproductive function including menstrual cycle (oligomenorrhea, amenorrhea, and normal cycle), ovulation and fertility, reproductive hormones (dehydroepiandrosterone, free androgens index, follicle-stimulating hormone, luteinizing hormone, SHBG, testosterone [TST]) (the units of measure were converted from ng/dL to nmol/L), The minor outcomes of interest were: metabolic parameters: homeostatic model assessment (HOMA-IR) and body composition (weight [kg], BMI, waist circumference [cm], and waist hip ratio).

*Risk of bias (quality) assessment:* We used the Cochrane risk of bias tool to evaluate the internal validity of included studies.^[27]^ This tool is based on 6 domains of study methods: sequence generation, allocation concealment, blinding, incomplete data, selective results reporting, and other sources of bias. Two authors (IKS, MCA) independently assessed each study using Covidence, then discussed final scores.

*Generalizability (external validity)*^[28]^: Two authors (IKS, PMSD) reviewed the included studies to identify the following factors: sampling frame, recruitment, and characteristics of study participants.

*Synthesis of findings:* Two authors (IKS, PMSD) reviewed the extracted data to synthesize results based on exercise intervention and type of exercise included in the intervention, for example, aerobic, resistance, and combined (aerobic and resistance) training. Two authors (IKS, GMS) 1st discussed the studies and outcomes to determine if it made clinical sense to combine data. We contacted the corresponding authors (n = 9) via email requesting additional information for the following outcomes: Ferriman and Gallwey score^[29]^; change in menstrual cycle; and fertility (pregnancy success rate). We received 4 responses from authors, who provided additional information.^[4,30–32]^

### 2.1 Risk of bias across studies

**Figure 2:**
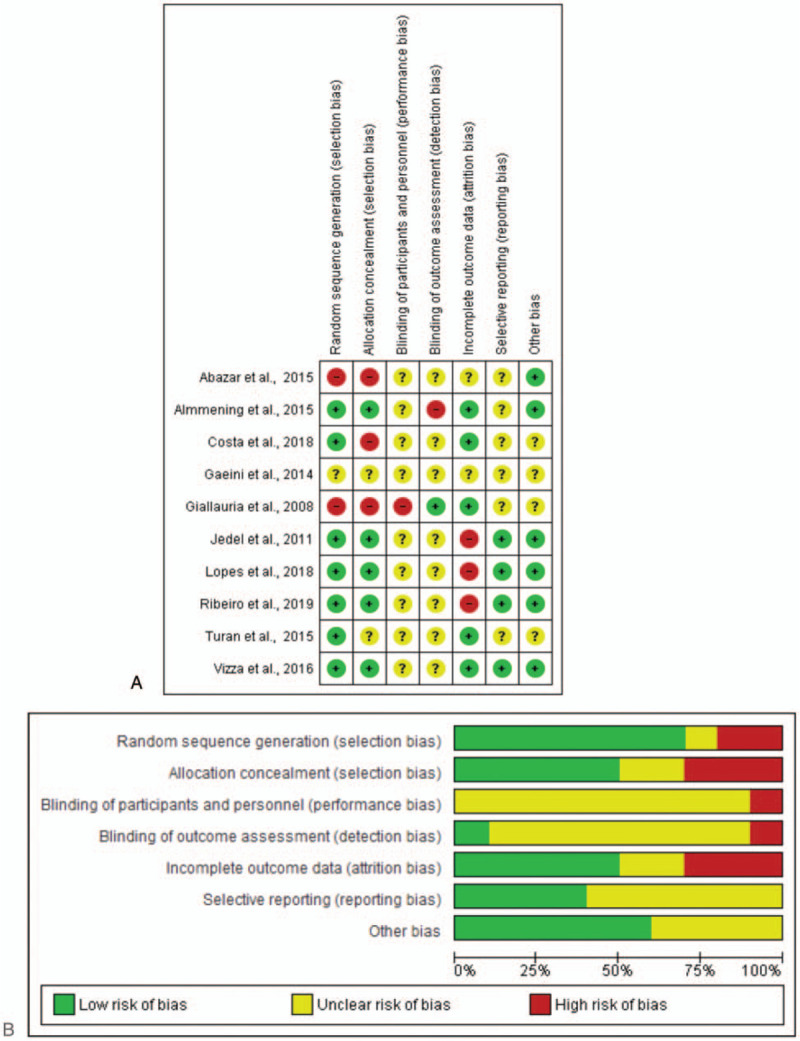
(A) Results of risk of bias assessment for individual level studies and (B) an overall summary for the included studies.

We created figures for risk of bias (Fig. 2A, B) for individual studies, and for the collective evidence. Two authors (IKS, RNC) also created and inspected funnel plots to determine publication bias. It was not appropriate to conduct a quantitative analysis for the risk of publication bias, because there were less than ten studies included in the systematic review.^[33]^

### 2.2 Ethical review

Ethical approval was not required for this systematic review as data were extracted from published evidence.

## 3 Results

We identified 1761 potentially eligible citations through electronic databases (5 additional citations were identified through manual searches of reference lists), which were reduced to 1238 after excluding duplicate articles. Of the 1238 total citations, 1144 were excluded at level 1. At level 2, 94 full text publications were reviewed, and 84 were excluded. In total, there were 10 articles included in the systematic review, and 8 articles in the meta-analysis (Fig. 1).

Characteristics of included studies: The included study characteristics are summarized in Table 2. Ten RCTs met the inclusion criteria^[4,10,30–32,34–38]^; studies were conducted at hospitals, clinical research centers, or at a university. The number of participants in each trial ranged from 13 to 124 (total n = 513) participants. The mean age of participants ranged from 22 to 30 years. Seven studies included PCOS diagnosis by the Rotterdam criteria,^[4,31,32,34,35,37,38]^ but 3 studies did not report diagnostic criteria.^[10,29,35]^ Most of the studies (9/10) reported their sample recruitment rates from 20% to 79% for all possible participants, and 37% to 89% of all eligible participants. Retention ranged from 84% to 100% participants. Four studies compared aerobic exercise with a control group (no intervention).^[10,32,34,36]^ One study compared aerobic exercise with electro-acupuncture and control group (receiving physical activity education).^[31]^ Two study compared continuous aerobic training vs intermittent aerobic training with a control group.^[37,38]^ Two studies compared combined (aerobic and resistance) training with a control group.^[30,35]^ Only 1 study compared high intensity aerobic exercise vs strength training with a control group (this group was recommended to exercise >150 minutes per week).^[4]^

**Table 2:**
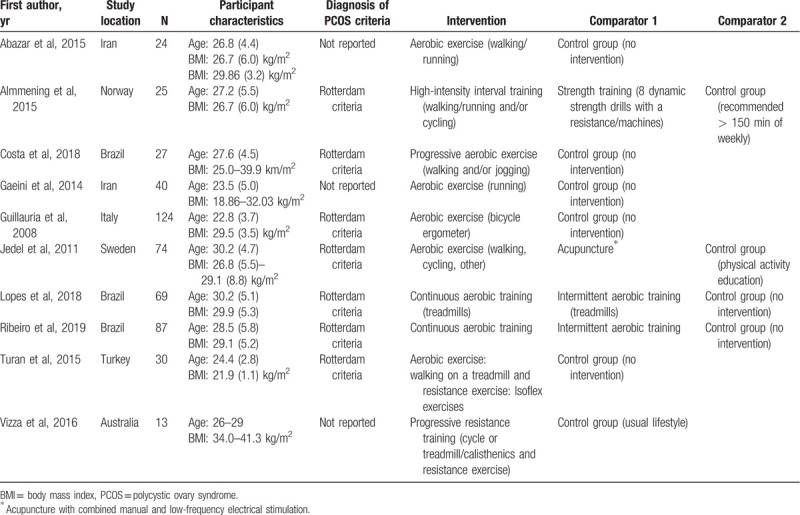
Characteristics of included studies (n = 10).

Table 3 describes the characteristics of the interventions included in the studies. Most studies included a 12- to 16-week intervention.^[4,10,30,32,34–38]^ Participants in the included studies performed aerobic exercise 3 times a week for 25 to 60 minutes per session.^[4,10,31,32,34–38]^ The intensity of exercise in most studies was moderate, with values ranging from 60% to 70% of maximal heart rate or VO_2_ max; the intensity was high (90–95%) in only 1 study.^[4]^ The effect of exercise interventions on reproductive function (e.g., menstrual cycle) was reported in 5 studies.^[4,30,35,36]^ Participants completed a standardized self-reporting menstrual diary, and 1 study collected basal body temperature (oral thermometer) and information via interviews.^[30]^ Menstrual cycle classifications included normal cycles, oligomenorrhea (35–42 days), and amenorrhea (primary and secondary [42 days to 6 months]).

**Table 3:**
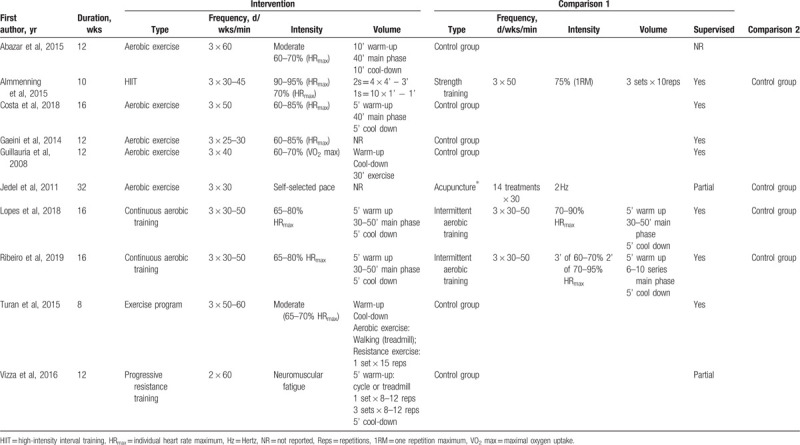
Characteristics of the types of interactions with training in included studies (n = 10).

*Risk of bias within studies:*Figure 2A, B present the bias risk assessment for the included studies. Most studies had unclear risk of bias, with the most common features not reported or missing being: blinding of participants, researchers, outcome assessors, or data analysts; and selective reporting. Allocation: Seven studies described adequate methods of generating random allocation sequence (via computer),^[4,30–32,35,37,38]^ while insufficient information was provided for the remaining studies. Five studies described adequate allocation concealment of both participants and researchers.^[4,30,31,37,38]^ Two studies did not provide enough information to determine a rating for allocation concealment,^[35,36]^ and 3 studies presented a high risk of bias.^[10,32,34]^ Blinding: In all studies, participants and researchers were not blinded to group allocation. Although this is commonly observed in exercise interventions, it can result in performance bias, favoring the treatment group. Researchers have more control over blinding of research team members who collect and analyze data, but only 1 study reported this information.^[34]^ Incomplete results data: Seven studies reported more than 20% (N = 91) of participants missing at final assessment, but they provided information on the number of participants who dropped out with reasons.^[4,30,32,34,35,38]^ Selective reporting: In 6 studies, there was no clinical trial registration, thus we were unclear about the selective reporting for these trials.^[4,10,32,34–36]^ Other potential sources of bias: Four studies reported insufficient information on study funding,^[32,34–36]^ and 6 studies were classified as low risk of bias.^[4,10,30,31,37,38]^

*Generalizability of findings:* All studies included women with PCOS. All participants had oligomenorrhea/amenorrhea and anovulation; the majority of participants were adult women aged 18 to 40 years and seemed to be representative of the target population.

*Effects of interventions:* We planned to synthesize results of all exercise interventions on reproductive function (hormones, menstrual cycle, ovulation rate, and fertility), but most studies only reported hormonal outcomes. However, because of high clinical and statistical heterogeneity between studies it was not appropriate to combine data to calculate an overall effect,^[39]^ and thus we can only provide a narrative synthesis for changes in menstrual frequency and classification (oligomenorrhea, amenorrhea, and normal cycle), ovulation, and fertility. Only 1 study noted changes in menstrual frequency after resistance training were reported in 3 women with PCOS (1 in the resistance training group and 2 in the control group)^[30]^ and three studies reported changes in the average menstrual cycle interval of some women participating in exercise interventions, but the results were not found in women in the control group.^[4,31,36]^ We conducted subgroup analyses comparing different types of aerobic and combined (aerobic and resistance) exercise training on hormones and body composition, and these data are presented below. However, due to lack of data, it was only possible to conduct meta-analyses on the effect of aerobic and combined training on reproductive function, metabolic parameters, and body composition.

### 3.1 Reproductive function (hormones)

**Figure 3:**
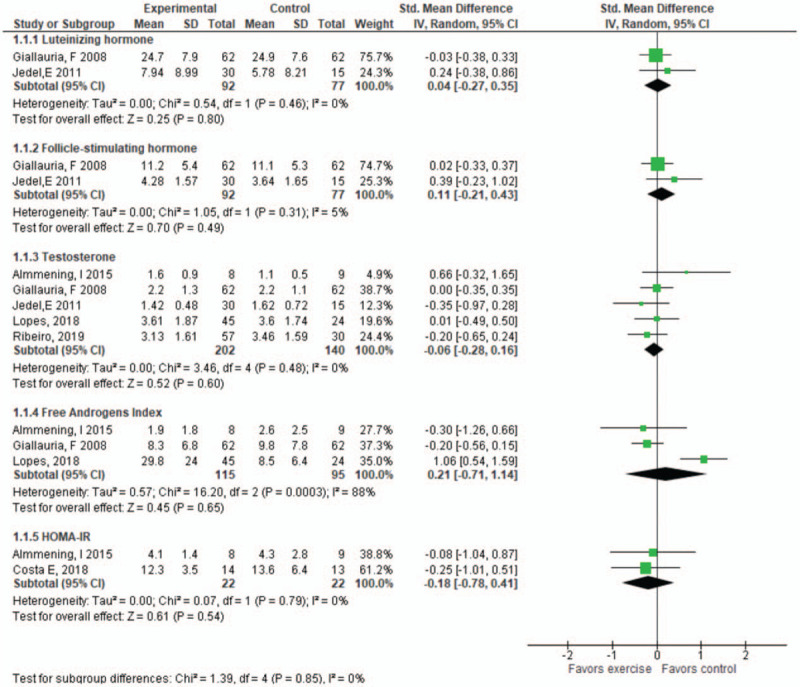
Change in hormones for aerobic exercise vs control group: luteinizing hormone, follicle-stimulating hormone, testosterone, free androgens index, and homeostatic model assessment (HOMA-IR). CI = confidence interval, SD = standard deviation.

*Aerobic exercise*: Six studies with 216 participants ^[4,31,32,34,37,38]^ objectively measured the effect of aerobic exercise on reproductive hormones and metabolic parameters from baseline to post-intervention. Details of the effect estimates, and GRADE ratings are summarized in Table 4. Compared with the control condition (no intervention received), there is low-certainty evidence that aerobic exercise makes little to no difference in luteinizing hormone levels (SMD 0.040, 95% CI −0.27, 0.35, *P* = .80) and follicle-stimulating hormone (SMD 0.11, 95% CI −0.21, 0.43, *P* = .49) in women with PCOS. Compared with the control condition (no intervention received), there is low-certainty evidence that aerobic exercise makes little to no difference in TST (SMD −0.06, 95% CI −0.28, 0.16, *P* = .60), free androgens index (SMD 0.21, 95% CI −0.71, 1.14, *P* = .65), and metabolic parameters with HOMA-IR (SMD −0.18, 95% CI −0.78, 0.41, *P* = .54) (Fig. 3).

**Table 4:**
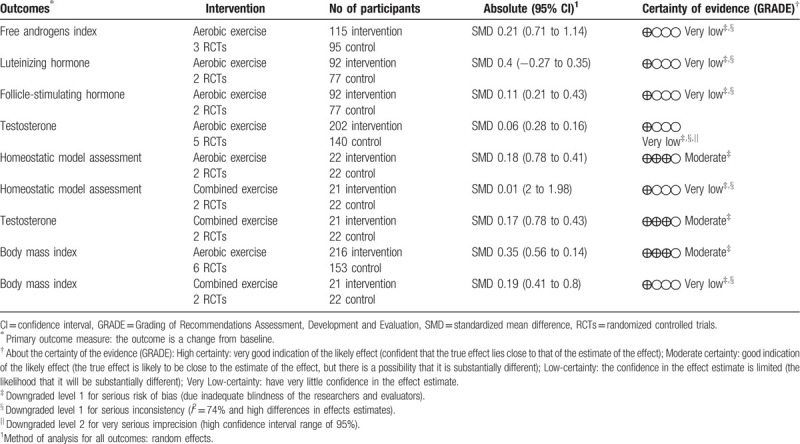
Summary of findings of clinical trials comparing the effects after 8 to 32 weeks of physical exercise on body and hormonal parameters of women with polycystic ovary syndrome.

**Figure 4:**
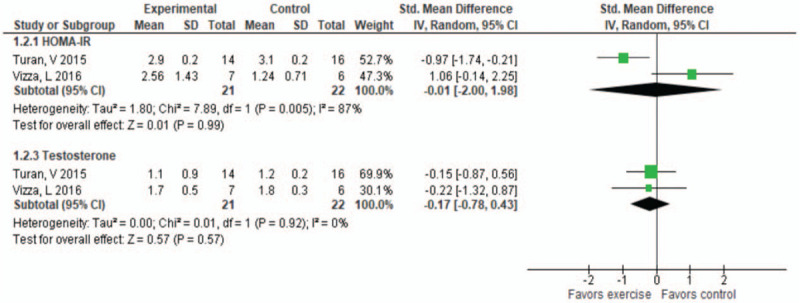
Change in hormones for combined exercise vs control group for homeostatic model assessment (HOMA-IR) and testosterone. CI = confidence interval, SD = standard deviation.

*Combined exercise*: Two studies with 29 participants^[30,35]^ measured the effect of combined exercise interventions on reproductive and metabolic parameters. Details of the effect estimates, and GRADE ratings are summarized in Table 4. Compared with control condition (no intervention received), there is a moderate certainty of evidence that combined exercise has little to no effect on TST (SMD −0.17, 95% CI −0.78, 0.43, *P* = .57) and HOMA-IR (SMD −0.01, 95% CI −2.00, 1.98, *P* = .99) (Fig. 4).

### 3.2 Body composition

**Figure 5:**
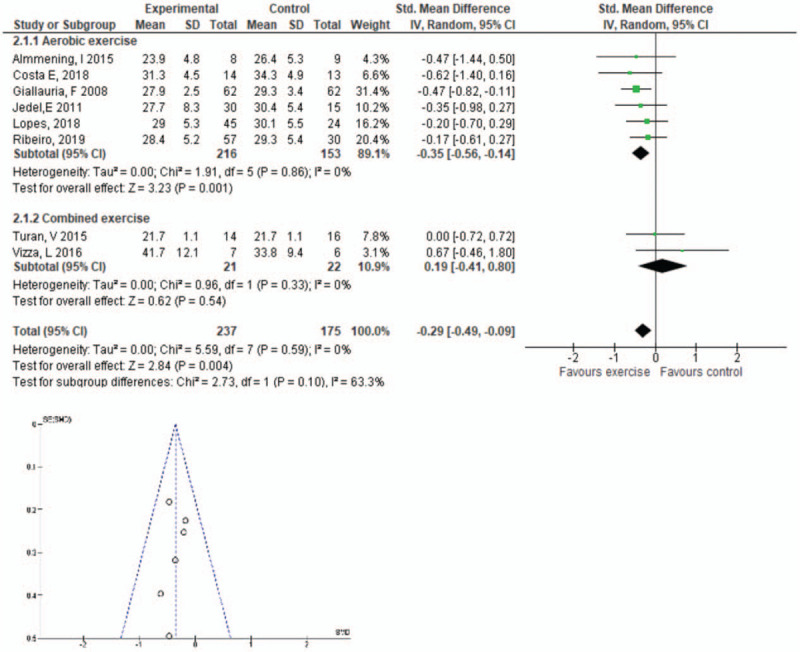
Change in body mass index (BMI) for aerobic exercise vs control group, and combined exercise vs control group and the funnel plot for exploring publication bias. CI = confidence interval, SD = standard deviation.

Eight studies with 237 participants^[4,30–32,34,35,37,38]^ (n = 216 in the aerobic intervention, and n = 21 in the combined exercise intervention) measured the effect of exercise on BMI. Compared with control condition (no intervention), there is moderate certainty evidence for lower BMI favoring the intervention group for aerobic exercise only (SMD −0.35, 95% CI −0.56, −0.14, *P* = .001), but there was low certainty that combined exercise had no effect on BMI (SMD 0.19, 95% CI −0.41, 0.80, *P* = .54). Publication bias was screened for in the funnel plot (Fig. 5).

## 4 Discussion

This systematic review identified 10 studies (including 533 women with PCOS), which tested the effect of exercise-based interventions on reproductive function and body composition. The majority of studies included relatively small sample sizes (average 32, range 15–124 participants): only 2 studies included 84 or more participants.^[31,34]^ The average duration of the interventions was 12 weeks (range 8–32 weeks): only 3 studies were longer than 12 weeks in duration.^[31,32,37]^ Seven studies had attrition rates higher than 20%, indicating the need for caution in interpreting the results.^[4,10,31,32,35,37,38]^ We were unable to conduct a meta-analysis on the effect of all exercise interventions on some major outcomes of reproductive function (menstrual cycle, ovulation, and fertility) due to the high heterogeneity between studies, and/or lack of available evidence. Most studies presented unclear risk of bias, and high risk of bias for blinding of participants, researchers, and assessors. Based on available data, there was insufficient evidence to draw conclusions about the overall effect of exercise on reproductive function. However, based on the subgroup analyses for exercise type, there was moderate certainty that aerobic exercise (10–32 weeks in duration) lowers BMI. As excess body weight can aggravate the underlying hormonal disturbances (such as increased levels of androgens and risk factors for cardiovascular disease and diabetes,^[11,13]^ this is an important clinical finding. Overall, these results serve to reaffirm the importance of exercise as a nonpharmacologic management strategy for the reduction of known risk factors for women with PCOS.^[39]^

In women with PCOS, increased BMI can exacerbate the metabolic manifestations, increasing the risk of cardiovascular disease and insulin resistance. These in turn play a key role in the central hormonal control of ovulation.^[40]^ Studies indicate that aerobic exercise improves several biomarkers related to health, and health organizations recommend exercise as a therapy for risk factors associated with obesity.^[37]^ The benefits that exercise exert on the health of individuals are well established.^[41]^ During aerobic exercise, the biochemical adaptations trigger a series of physiological stimuli that increase the oxygen uptake and oxidation of free fatty acids and circulate glucose as an energy source.^[42]^ In this way, aerobic metabolism is potentially increased to supply the energy required by muscle contractions, reducing body fat deposit, decreasing obesity rates, and improving cardiorespiratory fitness.^[43,44]^

We identified 4 published systematic reviews on the effect of exercise or lifestyle interventions (exercise and diet) for women with PCOS.^[15–18]^ These syntheses also reported positive effects of lifestyle interventions on reproductive health and body composition. However, our review extends this work^[15–18]^ by exploring the effect of exercise type. A 2nd unique feature of our review was the inclusion of GRADE recommendations. Here, we confirmed the effects of aerobic exercise on body composition, but in contrast, we noted a low certainty of evidence for little to no-effect of exercise on reproductive function (hormones). Taken together, the results suggest the combination of diet and exercise may be beneficial for clinical management of PCOS. Future trials could try to discern the contribution of diet and exercise, alone or in combination on health outcomes. Further, this review extends a recently published systematic review on the role of exercise in PCOS^[14]^ to emphasize future trials should report markers of reproductive function such as menstrual cycle and ovulation rate, either through menstrual history and/or ultrasound.

This systematic review has several limitations. First, there were only a small number of studies to include in this review. Second, most studies had small samples (15–40 participants), with the exception of one study (n = 124),^[34]^ and were of short duration. Third, some studies had methodological limitations (i.e., did not blind participants and/or assessors), which may have influenced the results. Fourth, the included studies involved participants with different BMI classifications; thus, this variability may have introduced more clinical heterogeneity, since the reproductive and metabolic characteristics may differ between women who are obese and overweight. In addition, 3 included studies did not clarify which methods were used for PCOS diagnosis and classification. Finally, there were few data on reproductive (major) outcomes, therefore we needed to rely on surrogate (minor) outcomes. Therefore, it was difficult to quantify the effect of exercise on our main outcome of interest. Thus, these limitations for the body of evidence must be considered when interpreting the results.

## 5 Conclusion

In conclusion, we note that there were few studies with small sample sizes, and with a relatively short duration that tested the effect of exercise on reproductive hormones and body composition in women with PCOS. Based on meta-analyses, there was low-certainty evidence of little to no effect of exercise alone on major reproductive outcomes; but moderate-certainty of evidence that aerobic exercise had a positive effect on body composition. Further, due to few available studies, it was difficult to explore the effect of exercise type on health outcomes. This review highlights the need for well-designed trials, of longer duration, testing the effect of specific exercise interventions on reproductive health outcomes to guide exercise prescription and clinical management. Finally, future studies should also investigate the effect of exercise and diet, alone and in combination, on the reproductive health outcomes of women with PCOS with different menstrual cycles and body composition.

## Acknowledgment

The Coordenação de Aperfeiçoamento de Pessoal de Nível Superior/Conselho Nacional de Desenvolvimento Científico e Tecnológico (CAPES/CNPQ) – Higher Education Personnel Improvement Coordination – for PhD scholarship Finance code 001.

## Author contributions

**Conceptualization:** Isis Kelly dos Santos, Maureen C. Ashe.

**Data cutarion:** Ricardo Ney Cobucci, Gustavo Mafaldo Soares.

**Supervision:** Maureen C. Ashe, Paulo Moreira Silva Dantas, Tecia Maria de Oliveira Maranhão.

**Writing – original draft:** Isis Kelly dos Santos, Maureen C. Ashe.

**Writing – review & editing:** Isis Kelly dos Santos, Maureen C. Ashe, Ricardo Ney Cobucci.

Isis Kelly dos Santos orcid: 0000-0001-7615-416X.
